# Recent Advances in 3D Printing of Aliphatic Polyesters

**DOI:** 10.3390/bioengineering5010002

**Published:** 2017-12-24

**Authors:** Ioana Chiulan, Adriana Nicoleta Frone, Călin Brandabur, Denis Mihaela Panaitescu

**Affiliations:** 1 Polymer Department, National Institute for R&D in Chemistry and Petrochemistry ICECHIM, 202 Splaiul Independentei, 060021 Bucharest, Romania; panaitescu@icechim.ro; 2 Symme3D and LTHD Corporation SRL, 300425 Timisoara, Romania; calin.brandabur@symme3d.com

**Keywords:** 3D printing, aliphatic polyesters, scaffolds, tissue engineering, polylactic acid, polyhydroxyalkanoates

## Abstract

3D printing represents a valuable alternative to traditional processing methods, clearly demonstrated by the promising results obtained in the manufacture of various products, such as scaffolds for regenerative medicine, artificial tissues and organs, electronics, components for the automotive industry, art objects and so on. This revolutionary technique showed unique capabilities for fabricating complex structures, with precisely controlled physical characteristics, facile tunable mechanical properties, biological functionality and easily customizable architecture. In this paper, we provide an overview of the main 3D-printing technologies currently employed in the case of poly (lactic acid) (PLA) and polyhydroxyalkanoates (PHA), two of the most important classes of thermoplastic aliphatic polyesters. Moreover, a short presentation of the main 3D-printing methods is briefly discussed. Both PLA and PHA, in the form of filaments or powder, proved to be suitable for the fabrication of artificial tissue or scaffolds for bone regeneration. The processability of PLA and PHB blends and composites fabricated through different 3D-printing techniques, their final characteristics and targeted applications in bioengineering are thoroughly reviewed.

## 1. Introduction

The diversity and complexity of materials expands continuously with a speed that is beyond of any expectations. Traditional manufacturing cannot meet all the requirements of the new products, especially when they are of small dimension and with high shape complexity. 3D printing, usually called “additive manufacturing”, is a useful tool for scalable fabrication of high complexity devices, or materials with multiple functions such as smart materials or customized products. It is very important in the process of prototyping and may also lead to the improvement of manufacturing by increasing the speed of production and lowering product cost. The first 3D printer was invented in 1987 and since then, this technology has grown rapidly because it brings multiple advantages over traditional production methods: (i) very complex structures can be created without added costs; (ii) the pieces are fabricated directly in assembled forms and the number of the components is consistently smaller compared to the same piece obtained by classical methods; and (iii) small series of personalized products can be obtained by this technique [1,2,3]. The interest for this technology is highlighted by the vibrant growth of the sales reported by 3D printer producers, who claim an increase of 17.4% in worldwide revenues, in 2016, as compared with previous years [4]. A substantial amount of research predicts the proliferation of this industry and a potential increase of the products and services from $6 billion in 2016 to $21 billion worldwide by 2021 [4].

3D-printing technology is attractive for many applications: (i) in the research field for prototyping or for a limited production of prototypes; (ii) in medicine to create 3D biomedical structures using digital models obtained with different medical imaging techniques (computer tomography, magnetic resonance imaging, ultrasound); (iii) in industry for prototyping and manufacture of spare parts for automotive, airplanes, etc. 3D-printing development is speeding up annually due to the reduction of the production cycles, waste, limited use of cutting fluids, and it becomes more accessible for small companies etc. [5]. Thus, 3D printing is often used to develop medical devices [6], flexible electronics [7,8], various pieces for automotive or robotics [9], art objects [2,3], precise replica of archeological objects [10] etc. Dental implants and porous scaffolds for tissue engineering, with increased surface roughness and improved mechanical performance and biocompatibility, used for bone fixation [11,12] are among the most studied medical devices.

The additive manufacturing methods are suitable for multiple types of materials, such as thermoplastics (acrylonitrile-butadiene-styrene (ABS), poly (lactic acid) (PLA), polyamide 6 (PA6), high-impact polystyrene, etc.), resins, metals (Al, steel, Au, Ag, Ti, alloys), gypsum-based powders, ceramics, waxed materials, biomaterials, paper, food. Polymers are by far the most used materials for 3D printing [13]. Likewise, aliphatic polyesters are among the most used biopolymers in the biomedical field due to their non-toxic, biodegradable and biocompatible character [14].

This mini-review deals with the use of aliphatic polyesters in 3D printing for medical applications, with a deeper attention on materials and methods suitable to construct scaffolds for tissue engineering. The industrial applications of 3D printing of aliphatic polyesters are quickly reviewed. The motivation behind this work resides from the recent scientific reports that highlight the ability of additive manufacturing to overcome the limitations of traditional methods such as molding, electrospinning, solvent casting, gas foaming, leaching etc. in the fabrication of medical products. Through 3D-printing techniques it is now possible to obtain superior control of the pore size, to manufacture scaffolds with complex architecture, and to implement biological functions in order to mimic the natural tissue [15,16]. A short presentation of the main 3D-printing methods will be followed by an overview of two most important classes of thermoplastic aliphatic polyesters, poly(lactic acid) and polyhydroxyalkanoates (PHA), their blends and composites that were processed by these methods. Finally, a discussion of the future perspectives and research approaches is included.

## 2. Short Overview of the Main 3D-Printing Techniques

**Figure 1:**
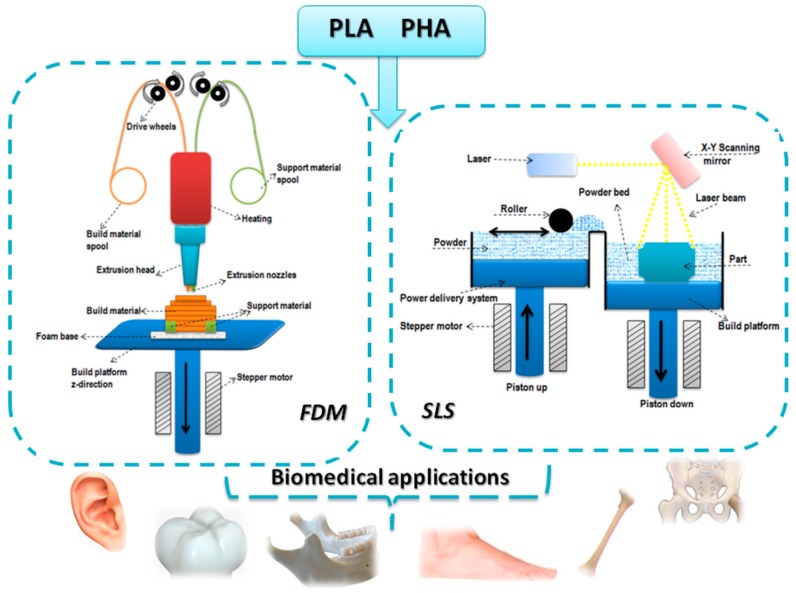
3D-printing techniques employed for PLA and PHA.

Broadly, the main 3D-printing techniques commercially available are: (i) selective layer/laser sintering (SLS); (ii) fused filament fabrication (FFF), also known as fused deposition modeling (FDM, trademark of Stratasys) or molten polymer deposition; (iii) stereolithography; (iv) digital light processing; (v) polyjet / inkjet 3D printing and (vi) electronic beam melting [3,16]. Only SLS and FDM have been used for the 3D printing of aliphatic polyesters (Figure 1).

### 2.1. Selective Laser Sintering

In SLS technique, the 3D-designed model is transferred to the printer, where an infrared laser beam fuses the polymeric powder, especially polyamides and thermoplastic polyurethanes (TPU), as well as metal and ceramic powders, into thin layers, one layer at a time [17]. After the completion of a layer, a new layer of powder is applied to it and then subjected to another round of heating action and sintering. The process is repeated and the completed object is removed from the printer, brushed and sandblasted in order to remove any trace of powder [18]. Depending on the application and material used, the printed object can be further polished and/or dyed. This technique is characterized by a high resolution, is suitable for functional polymers and does not require a support material or structures, so the printed structures can be used without further cleaning steps [19]. Polyamide 12 (PA12) or its powdered blends with PA6 were successfully printed through SLS and represents around 90% of the total industrial consumption [20]. Other materials processed to a much lesser extent through SLS are polyamide 11, PLA and polyether ether ketone.

### 2.2. Fused Deposition Modeling

**Figure 2:**
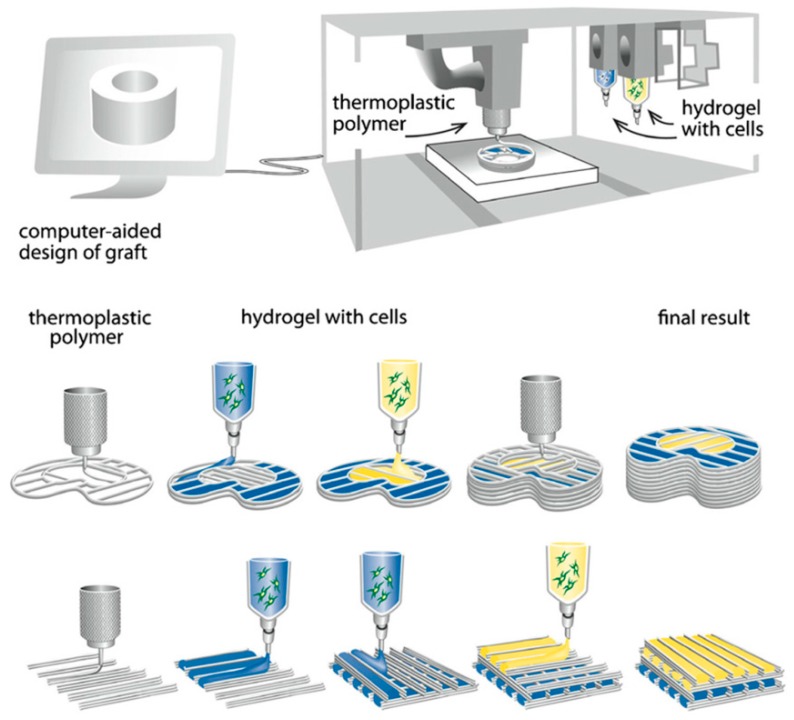
FDM schematic of the bioprinting of tissue and organs.

Through this technique, filaments made of thermoplastic materials are extruded in thin threads and deposited layer by layer in the desired 3D structure and adhere to each other by physical interactions. The layer underneath hardens as it cools and binds with the new layer that is added on the top, remaining a fully solidified structure throughout the process. FDM is already used to produce commercial plastics and, in general, is the most used among all the techniques; this is partially because of the low price of the printer and the facile manipulation, which makes it possible even for home use. Thermoplastic polymers currently processed with FDM are ABS and PLA. Other polymers were also found suitable for this technique: acrylonitrile-styrene-acrylate, PA12, polycarbonate, polyethylene terephtalate, TPU and thermoplastic elastomers. The roughness of the 3D-printed structures is an important issue in the case of FDM, since it affects not only the appearance but also the mechanical resistance of the products. A polishing device connected to the 3D printer [21], the use of the vaporized acetone to melt uniformly the surface of 3D-printed prostheses made of ABS [22] and filling the grooves with the material dissolved by the solvent stored in a pen-style device [23] were among the solutions proposed to remove the layer grooves. FDM technology also allows the printing of cells suspension into a scaffold support. A schematic illustration of a tissue-engineered structure obtained by FDM bioprinter is presented in Figure 2.

## 3. Aliphatic Polyesters for Additive Manufacturing 

Well selected and up-to-date information on the additive manufacturing of various polymers were recently reported [13]. Considering the huge importance of aliphatic polyesters for biomedical applications, this review gives thorough information on the use of 3D-printing techniques in the case of PLA and PHA, correlated with the properties of the manufactured products and their applications in bioengineering.

### 3.1. Poly(Lactic Acid)

**Figure 3:**
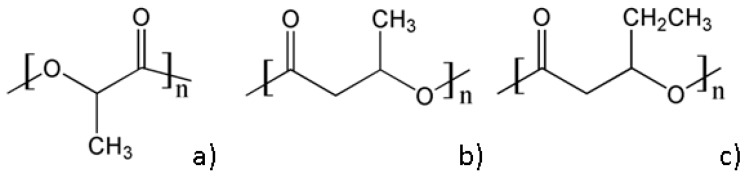
Chemical structures of PLA (**a**), PHB (**b**) and PHV (**c**).

PLA is up to now the most used bioplastic for 3D printing by FDM, intended to be used in regenerative medicine, mostly as scaffolds for tissue engineering. PLA is thermoplastic aliphatic polyester (Figure 3a) prepared from fossil fuels or derived from renewable resources such as cornstarch or sugarcanes, rendering it accessible and inexpensive. PLA properties are strongly influenced by even small amounts of enantiomeric impurities. Pure poly(l-lactic acid) (PLLA) or poly(d-lactic acid) are semicrystalline polymers with a glass transition temperature (*T_g_*) around 57 °C and a melting temperature of about 175 °C while PLA with a content of 50–93% l-lactic acid is completely amorphous [24]. Generally, amorphous grades have better processability and wider processing window than the crystalline grades [25] but much lower mechanical properties (Table 1). *T_g_* value is important for amorphous PLA because it determines the maximum usage temperature in most applications while both *T_g_* and *T_m_* vales are important in the case of crystalline PLA applications. Some thermal and mechanical characteristics of PLA are given in Table 1.

PLA is the most studied aliphatic polyester for biomedical and packaging applications, due to its biocompatibility, biodegradability, clarity, high mechanical strength and modulus, and facile processability through extrusion, injection molding or casting [14]. Moreover, its lower coefficient of thermal expansion and non-adherent properties to the printed surface makes PLA a suitable material for 3D printing. In addition, it is already approved by the Food and Drug Administration (FDA) and European Medicines Agency (EMA), which makes it suitable for rapid transfer from production to clinical trials and fabrication of medical devices, pharmaceutics or various consumer products [26]. This material was intensively studied for applications such as sutures, scaffolds, extracellular matrix, dental implants, drug delivery systems, cell carriers, bioresorbable screws for bones fractures, bioabsorbable meniscus repair and stents, hernia meshes, to name just a few [27].

To date, the most common technique for 3D printing of PLA is fused deposition modeling [12,33,34,35,36,37,38,39,40,41,42,43,44,45,46,47,48,49,50,51]. Printing parameters such as build orientation, layer thickness, raster angle, raster width, air gap, infill density and pattern, feed rate and others directly influence the quality and the mechanical properties of the FDM printed parts [35]. Considering the importance of mechanical performance for the printed parts, the majority of current studies are focused on the influence of printing parameters on the mechanical properties of the resulted parts [33,34,35]. Therefore, many recent studies highlighted the mechanical and biocompatibility characteristics of PLA or its composites after 3D printing [36,37,38,39,40].

#### 3.1.1. 3D Printing of PLA through Fused Deposition Modeling

A detailed study comparing the mechanical response of 3D-printed PLA blocks versus that of injection-molded PLA was provided by Song et al. [33]. PLA filament (commercial, diameter 1.75 mm) was deposited in a single direction using FDM method. Specimens cut from the printed blocks were measured along different material directions. 3D printing had a limited influence upon material elasticity; both axial and transverse stiffness being similar to that of injection-molded PLA while the inelastic response of the 3D-printed material was ductile and orthotropic. It was observed that the fracture response of the 3D-printed product was tougher when loaded in the extrusion direction than in the transverse direction. Moreover, the unidirectional 3D-printed material showed an increased toughness as compared to injection-molded PLA, due to its layered and filamentous nature. By controlling the process parameters (extruder temperature, extrusion speed, and deposition speed during 3D printing) the porosity of the material can be controlled.

Other authors used a custom 3D-printing profile for printing the specimen entirely in a single raster orientation in order to evaluate the connection between printing orientation and the material anisotropy [34]. It was found that the 45° raster orientation resulted in a slight improvement of the ultimate tensile strength and fatigue endurance limit as compared to the specimens printed at 0° and 90° raster orientation angles. Still, the mechanical properties of printed specimens were similar to those of PLA filament.

In addition to mechanical properties, 3D-printing process parameters have also great influence on the shape-memory properties of the printed parts, as reported by Wu et al. [26]. Authors used orthogonal experimental design method in order to evaluate the influence of four FDM parameters (layer thickness, raster angle, deformation temperature and recovery temperature) on the shape-recovery ratio and maximum shape-recovery rate of 3D-printed PLA. Authors concluded that the shape-memory effect of 3D-printed PLA parts depended more on recovery temperature and less on the deformation temperature and 3D-printing parameters. These findings could be of great interest for biomedical applications (self-expanding vascular stents, the elimination of thrombus) as well as the selection of parameters for 4D printing.

The possibility to replace conventional processing technique with additive manufacturing is considered by most to be unrealistic and the reasons for this opinion come from some drawbacks of the latter, such as the impossibility of manufacturing very large objects, the limitation to a small range of materials and the cost of high-performance 3D printers. This cost is subsequently reflected by the price of the final product. In order to evaluate the cost of the 3D procedure and the possibility to reduce it, Chacón et al. tried to find a connection between printing parameters and the FDM manufacturing cost [35]. Thus, PLA samples were obtained from a filament with a diameter of 1.75 mm using a low cost desktop 3D printer. Build orientation, layer thickness and feed rate parameters were analyzed and it was found that printing time decreases as layer thickness and feed rate increase. Thus, the manufacturing cost is directly related to the layer thickness and feed rate parameters.

It has been shown previously that it is possible to control the mechanical properties of PLA printed parts using an optimal selection of FDM parameters but other properties are also of great importance when referring, for example, to biomedical applications. In this respect, recent studies focused on the evaluation of PLA printed parts for reconstructive surgery and tissue engineering [36,37,38,39]. In a paper by Wurm et al. [39] FDM was successfully employed for the fabrication of PLA discs and the influence of processing technique upon biocompatibility of printed parts was assessed. In vitro tests, using human fetal osteoblasts showed no cytotoxic effects of PLA discs. Since FDM proved no negative influence on the biocompatibility of PLA, this 3D-printing technique could be further used in the reconstructive surgery for the production of individual shaped scaffolds or other implants. The filaments were printed at a nozzle temperature of 225 °C, which led to an enhanced degree of crystallinity of 22% and, finally, to a modulus of elasticity of 3.2 GPa that fits the requirements for maxillofacial implants [39].

PLA membranes, with a thickness of 100 µm and pores diameter of 200 µm, were also fabricated by direct 3D-printing method, using a PLA chloroform solution, of 5%, well dissolved by heating at 45 °C, for 24 h [40]. The PLA membranes were further seeded with human osteoprogenitors and endothelial progenitor cells and then assembled one above the other, to form a layer-by-layer (LBL) structure. After evaluating the properties of LBL constructs in vitro, in 2D – 3D, the authors stated that LBL approach could be suitable for bone tissue engineering in order to promote cells proliferation and a homogenous distribution into the scaffold.

The surface roughness of the 3D structure is very important, since cell attachment and proliferation are mainly influenced by the surface tension, roughness and stiffness of the substrate [41]. In order to enhance the roughness of the surface, Wang et al. used cold atmospheric plasma (CAP) to treat a 3D-printed PLA scaffold fabricated using a FDM printer [12]. They obtained an increase of roughness from 1.20 nm to 27.60 nm upon exposure to CAP for 5 min as compared to the untreated PLA scaffold. A significant increase of the hydrophilicity, revealed by a decrease of the contact angle from 70° to 24°, was obtained after the CAP treatment, which was proven to be a facile route to positively impact the proliferation of the osteoblasts on the PLA scaffold.

**Figure 4:**
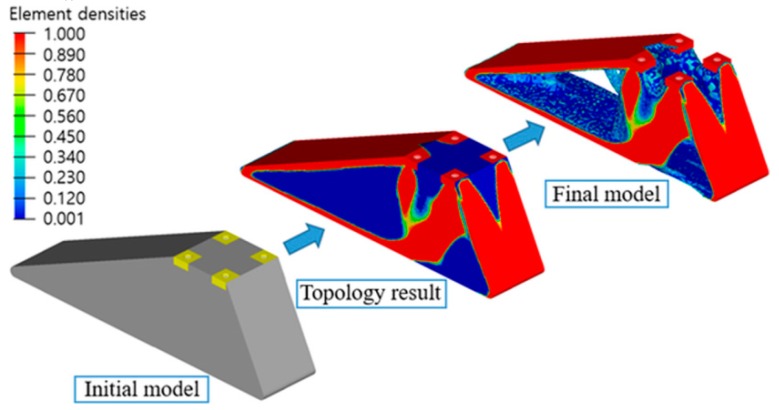
Topology optimization process of designed prosthetic foot. Reproduced with permission from [42].

Another research study proposed a design process for FDM 3D printing of a prosthetic foot made from PLA which can significantly reduce the prosthetic weight, design and manufacturing cycle [42]. Through this process the initial model was optimized using topology optimization methods. The optimized model was printed directly from a 3D desktop printer. The authors obtained a reduction of the prosthetic feet weight by 62% compared to the initial model and a more accurate 3D-printed product (Figure 4). The proposed method facilitates the manufacturing process and reduces the fabrication time, by skipping the transfer to computer-aided design software. This research can contribute to the improvement of the quality of life of patients who need foot-customized prostheses.

Flores et al. also emphasized the cost effectiveness, easy manufacturing and high accuracy of the 3D-printing technology. They successfully obtained auricular prosthesis, fully customizable, which replicate in an astonishing degree the skin color and texture of the patient. However, further maintenance and potential replacement of this 3D ear prosthesis may convince the patient to agree with other alternative options [43].

#### 3.1.2. 3D Printing of PLA Composites through Fused Deposition Modeling

PLA has relatively low glass transition temperature (55–60 °C), low toughness and weak heat resistance, which limits its application. Scaffolds made only of PLA do not mimic sufficiently the native bone architecture and they do not ensure properly the cell colonization or mechanical properties. For some uses, PLA needs to be mixed with other polymers or fillers in order to create materials with improved thermal and mechanical properties, or higher biocompatibility for biomedical purposes.

**Figure 5:**
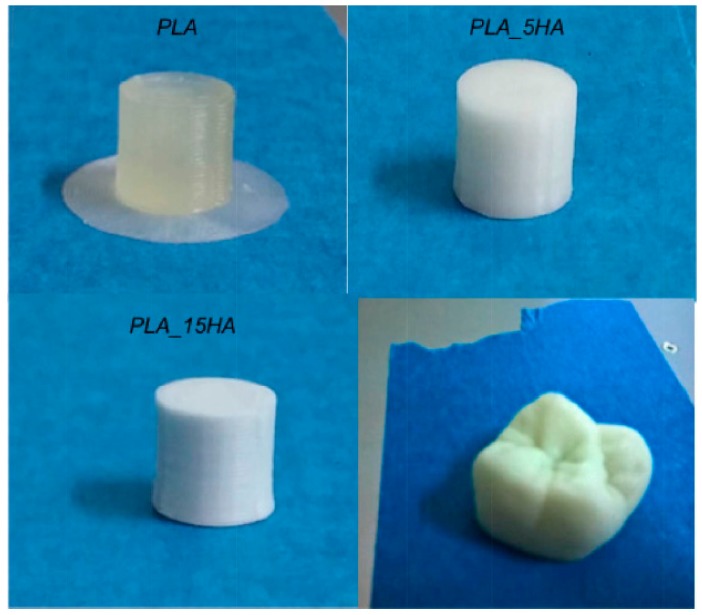
PLA/HA nanocomposites by FDM 3D printer. Reproduced with permission from [48].

Good improvement of properties was achieved by adding 15 wt.% of nano-hydroxyapatite (HA) to PLA [47,48]. The composite was extruded in filaments and then 3D printed at a nozzle temperature of 220 °C [47]. Long-term creep test revealed a superior hardness of the 3D-printed composite as compared with PLA scaffold and consequently an increase in creep resistance. However, both samples displayed identical delamination destruction, due to limitations of the 3D-printing technique that cannot ensure completely sinterization between the layers. This causes the air to be trapped between layers, which lead to creation of voids. As expected, in vivo tests made on mice showed no inflammatory reaction even after 2 months and a slow biodegradation rate. Corcione et al. used filaments made of PLA and HA in different concentrations to obtain a molar tooth (Figure 5); this was successfully printed using FDM [48].

No noticeable difference was observed for both composites in terms of morphology, thermal behavior and crystallinity. A good dispersion of the filler was observed, but some expectable agglomerations of the nanoparticles took place, both at 5 and 15 wt.% HA. Similar values of the glass transition temperature and crystallization degree were obtained for PLA and PLA/HA samples. The addition of 15% HA influenced the rheological behavior by a significant increase of viscosity and the mechanical properties by the increase with almost 4% of the average compressive modulus as compared with the PLA sample.

Zhuang et al. used 3D printing to obtain plastic items with anisotropic heat and resistance distribution, which allows storing a simple message as color information in the printed objects. These were obtained from conductive graphene doped poly(lactic acid) (G-PLA) [50]. The authors used a method of programmed mixed printing to manufacture PLA composites with anisotropic properties. They stated that the method could be applied to other polymeric materials for a wide range of applications including biomedical ones.

For some medical applications, the rigidity and brittleness of the PLA are undesirable and the addition of elastomers is the easiest solution to overcome this drawback. TPU are among the most used polymers in 3D printing. They are also attractive for some biomedical applications due to their biocompatibility, high elongation at break and good abrasion resistance. As shown before, the use of different fillers impart to PLA exceptional mechanical strength, electrical conductivity, and enhanced thermal stability. Among them, composites with carbon fibers and graphene oxide (GO) proved to be also suitable for 3D-printing process. The addition of GO and TPU may have a cumulative effect of increased flexibility and mechanical strength. Chen et al. studied both the influence of the GO concentration and printing orientation on the mechanical properties of a TPU/PLA (7/3) blend [51]. Compression modulus tests have shown an increasing trend with the increase of GO content from 0.5 to 5 wt.% for both printing orientations, but the highest values were found for the specimens having the same printing orientation and height direction. The addition of only 0.5 wt.% GO determined an increase of the tensile modulus by 75% as compared with TPU/PLA sample, further addition of nanofiller determining a reduction of properties. This was explained by the percolation effect, which appeared below 2 wt.% GO content. All TPU/PLA/GO scaffolds supported fibroblast cells growth and proliferation, with the optimum effect at 0.5 wt.%.

#### 3.1.3. 3D Printing of PLA and PLA Composites through SLS

An important requirement for the powders intended for SLS is the sintering behavior, which is greatly influenced by the thermal properties, melt viscosity, melt surface tension, and powder surface energy [13]. Semicrystalline polymers such as PLA exhibit a large change in both viscosity and density within a narrow temperature range upon melting and crystallization, which affects their processing through SLS method. Therefore, the consolidation of semicrystalline powders is conducted by local heating to temperatures slightly above melting temperature [13].

Thus, a porous scaffold was sintered from PLLA using a modified commercial Sinterstation^®^ 2000 system (3D Systems, Valencia, CA, USA), adapted for the use of small amount of raw material [52]. The PLLA was in the form of microsphere of 5–30 µm in diameter, obtained by oil-in-water emulsion solvent evaporation technique. The SLS was conducted at 15 watts, the PLLA powder bed was preheated at 60 °C and the scan spacing was 0.15 mm. The control of the 3D scaffold porosity was difficult, since PLLA microspheres were partially melted and entangled, as revealed by SEM images [52].

The same equipment was further used to manufacture scaffolds made of PLLA and carbonated hydroxyapatite (CHAp) nanospheres, intended for bone tissue reconstruction [52,53,54,55]. Both PLLA microspheres and the PLLA/CHAp nanocomposite with 10 wt.% CHAp were prepared by emulsion method. The good dispersion and embedment of the CHAp nanoparticles in the PLLA matrix conducted to the increase of nanocomposite hardness, as revealed by the nanoindentation test. The SLS processing parameters (laser power, scan spacing, part bed temperature, roller speed, scan speed) were optimized in order to obtain adequate porosity, good compression properties, osteoconductivity and biodegradability of the PLLA and PLLA/CHAp scaffolds. The addition of the CHAp was found to influence the thermal behavior, by lowering the glass transition temperature and cold crystallization temperature and increasing to a lesser extent the melting temperature of PLLA. CHAp addition favored the powder deposition but reduced the fusion degree compared with pure PLLA powder. The porosity was mostly influenced by the part bed temperature, being enlarged in the case of nanocomposite [53,54,55].

Duan et al. reported the fabrication of PLLA/CHAp nanocomposite scaffolds with controllable architecture and pore size for bone tissue engineering starting from PLLA microspheres and PLLA/CHAp nanocomposite microspheres through SLS method [56]. Both raw CHAp microspheres and nanocomposite microspheres were made “in house” using a nanoemulsion method in the first case and double emulsion solvent evaporation method in the second case. More than that, in order to ensure a firm foundation and to facilitate handling of the sintered scaffold a solid base was incorporated into the scaffold design. The sintered PLLA/CHAp nanocomposite scaffolds exhibited a lower porosity value (66.8 ± 2.5%) as compared with the control PLLA scaffolds (69.5 ± 1.3%). The mechanical response (the compressive strength and modulus) of 3D scaffolds under dry conditions was higher than the one obtained under wet conditions (immersion in phosphate-buffered saline at 37 °C). In terms of biological evaluation, the PLLA/CHAp nanocomposite scaffolds exhibited a similar level of cell response compared with control PLLA scaffolds. After 7 days culture, the human osteoblastic cells were found to be well attached and spread over the strut surface and interacted favorably with all scaffolds [56].

#### 3.1.4. Other Directions in 3D Printing of PLA Based Materials 

PLA may also fit the requirements for electronic devices and other fields by chemical modification or by the addition of different fillers and polymers [44]. The presence of ionic liquids (IL) in a PLA 3D-printed structure provides unique features to PLA-based electronics; IL were recently added in the process of additive manufacturing of PLA filaments by Dichtl et al. [45]. The mixture was prepared by simply adding IL (5 and 10 wt.%) into a PLA chloroform solution, stirring for 12 h and then casting on a teflon plate. A significant enhancement of the PLA conductivity was noticed after the addition of trihexyl tetradecyl phosphonium decanoate, but further mechanical investigations are required to certify that this mixture is suitable for different applications. Prashantha and Roger studied the mechanical and electrical properties of 3D-printed specimens made from commercially available PLA filaments filled with 10 wt.% graphene [46]. The porosity distribution of the structure and the adhesion between layers were characterized through X-ray computed tomography. The results suggested that a shorter deposition time is favorable to obtain better interactions between the fused filaments and the maximum concentration for a suitable graphene dispersion is 10 wt.%. The increase of the electrical resistivity of the 3D-printed specimens, compared with the same composite before FDM processing, was explained by the alignment of the graphene nanoplatelets in the same direction with the deposited filaments. The reinforcing effect of graphene was highlighted by the increase of the storage modulus with more than 20% and tensile strength with 27%, with respect to PLA, as revealed by the DMA and static mechanical analysis [46].

PLA reinforced with 15 wt.% short carbon fibers (length about 60 mm) was manufactured by 3D printing based on fused filament fabrication and tested for mechanical and morphological properties [49]. The PLA composite showed a higher increase in stiffness in the direction of printing. This behavior was explained by the morphological results, which revealed that the short carbon fibers were mostly aligned with the length of the 3D-printing filament, and remained aligned with the direction of printing within the PLA composite.

Wood pulp fibers (WPF) are valuable reinforcements for many polymers but the application of FDM technology for 3D printing of biocomposites with WPF is a difficult process [57]. The issues are related to the low thermal degradation temperature of the fibers, the small size of the nozzle used in FDM process and the poor dispersion of the fibers in the hydrophobic matrix, which causes fibers accumulation in the nozzle. A full enzymatic treatment was used to modify the surface of thermomechanical pulp (TMP) fibers [57]; TMP fibers modified via laccase-assisted grafting of octyl gallate (OG) showed improved interfacial adhesion with PLA and a remarkable impact on the mechanical properties of PLA-TMP fibers composites. Moreover, filaments obtained from PLA reinforced with OG-treated fibers showed a good behavior during the 3D printing [57].

3D printing of a recycled PLA composite has proved to be a viable solution to the environmental issues, since the remanufactured 3D structure showed even better mechanical properties than the original one. Tian et al. have managed to recover a PLA/carbon fiber composite in a 100% rate for the carbon fiber and 73% for PLA matrix. They reused the material for the fabrication of new filaments, with a carbon fiber content of 10 wt.%, that were further processed by 3D printing [58]. No increase of the tensile strength was observed for the remanufactured composites as compared with the original composite, but other representative characteristics were improved, such as flexural strength, which increased with around 25%. The aging process of the PLA matrix was impossible to be avoided due to repeated thermal cycles, but the mechanical performances were maintained by the addition of pure PLA in the 3D printing of the recycled composite.

### 3.2. Polyhydroxyalkanoates

The polyesters of aliphatic hydroxyacids, PHA, are natural polymers with some of their properties similar to those of conventional plastic materials but, in addition, they show biodegradability and biocompatibility. PHA are biosynthesized intracellularly as spherical inclusions by some bacterial strains in unbalanced growing conditions (low concentrations of nitrogen, phosphorus, oxygen or magnesium and an excess of carbon). Depending on the number of carbon atoms in the lateral chain, they may be brittle materials or elastomers. Both types are interesting materials for the biomedical field, especially for scaffolds and implants.

Short-chain-length PHA contain 3–5 carbon atoms and show high stiffness and brittleness in relation to their high crystallinity (50–80%) [24]. Poly(3-hydroxybutyrate) (PHB) (Figure 3b) and poly(3-hydroxybutyrate-co-3-hydroxyvalerate) (PHBV) are by far the most studied of PHA and are commercially available. PHB is biodegradable and biocompatible and can be processed with common plastic manufacturing equipment. However, its brittleness and small processing window limits its applications. PHBV, obtained by copolymerization with hydroxyvalerate (HV), is a more ductile material, with lower melting point and decreased strength and stiffness [24,59]. The properties of PHB or PHBV strongly depend on the processing conditions and composition (Table 2).

Cell attachment and viability tests were performed using various cultures and revealed a good biocompatibility of PHA to these cells. For example, CHL fibroblast cells showed good adhesion and proliferation on PHB scaffolds [60]. Moreover, polyhydroxyalkanoates degrade into non-toxic oligomers being suitable candidates for in vivo use in medical applications.

However, the reconstruction of some parts of the human body and organs using PHA is a very complex and difficult process because of the large differences between patients. The patient specific anatomical data should be considered for reconstruction and 3D printing is a promising technique to produce complex medical devices according to the computer aided design of the damage part or organ. Only few data were reported regarding the application of rapid prototyping techniques (RP) for the fabrication of PHA scaffolds [63,64,65,66,67,68,69,70]. Comparing to PLA, PHA cover a much broader range of properties and, therefore multiple possibilities of 3D printing.

#### 3.2.1. PHA Filaments for Fused Deposition Modeling

PHA filaments can be used to obtain scaffolds by using FDM. Wu et al [63] obtained PHBV/palm fibers (PF) composite for 3D printers by melt mixing PHBV grafted with maleic anhydride (PHBV-g-MA) and silane treated PF. The filaments (diameter 1.75 ± 0.05 mm) were obtained from these composite materials by extrusion at 130–140 °C and 50 rpm [63]. The treatments ensured a better adhesion at polymer–filler interface and avoided the phase separation and fluctuation in the filaments diameter. The treated composites showed enhanced mechanical properties compared to that of PHBV matrix and untreated composites and higher biodegradation rate than that of PHBV when incubated in soil. Increased tensile strength and antibacterial activity were also reported for PHBV-g-MA/wood flower (WF) composites prepared with the same purpose, for 3D-printing filaments [64]. Thus, the tensile strength of PHBV-g-MA/WF composites was 6–18 MPa greater than that of untreated composites and increased with the increase of WF content [63]. Wu and Liao [65] have also prepared 3D-printing filaments from PHBV-g-MA composites with acid oxidized multi-walled carbon nanotubes (MWCNTs) using a similar method. Highly improved thermal stability, Young’s modulus and antibacterial activity were obtained for only 1.0 wt.% MWCNTs in PHA-g-MA matrix [65]. However, no study on the behavior of these types of filaments in a real 3D-printing process was reported.

#### 3.2.2. PHA Structures Obtained by Selective Laser Sintering

##### 3.2.2.1. SLS Applied to Pure PHB 

**Figure 6:**
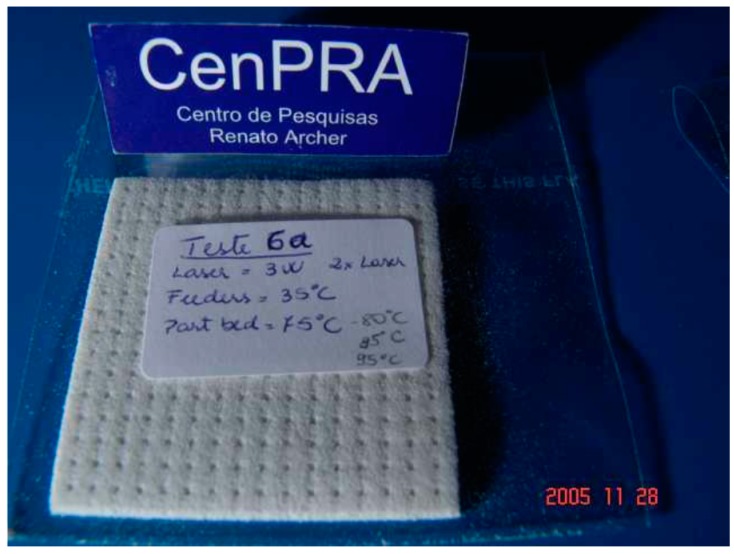
Sintered PHB using SLS containing pores of 1 mm in diameter [66].

SLS technique is very attractive because porous structures with very controlled pore size may be built up without the need of any additives such as plasticizers. Preliminary RP tests with a polyhydroxyalkanoate were done by Oliveira et al. using SLS technique [66]. They worked with a polyhydroxybutyrate powder in pure form (without additives) and obtained structures of about 2.5 mm in thickness (up to 10 layers) with 1 mm holes by SLS (Figure 6).

They also reported the difficulties encountered with the application of SLS technique to PHB powder, such as excessive dust drag, curbing of the coating or release of vapors and the solutions adopted to solve these problems [66].

Pereira et al synthesized porous 3D cubes with orthogonal channels measuring 0.836 mm in diameter by SLS, starting from a poly(3-hydroxybutyrate) powder from PHB Industrial S/A (Brazil) [67]. A thin layer of powdered PHB was scanned by a CO_2_ laser and sintered, the polymer layers being deposited one on the top of each other until the object reached the dimensions of the virtual model. The obtained objects showed geometrical and dimensional features closed to the model [67]. No significant change in crystallinity, glass transition, melting or crystallization temperatures of PHB were detected after SLS process, suggesting no thermal degradation [67]. Moreover, the possibility to recycle PHB through 3 rounds of SLS processes without any sign of degradation was also demonstrated.

##### 3.2.2.2. SLS Applied to PHA Nanocomposites

One of the most studied applications of PHA based materials is in bone tissue engineering. PHA nanocomposites were designed to obtain 3D scaffolds that mimic the structure and function of an extracellular matrix (ECM) and support cells adhesion and proliferation [56,68,69,70]. Thus, bionanocomposites microspheres from PHBV and nano-sized osteoconductive inorganic fillers were obtained using a solid-in-oil-in-water emulsion/solvent evaporation method [56]. Nano-sized calcium phosphate (Ca-P) was prepared for this purpose and dispersed in a PHBV-chloroform solution by ultrasonication to form a solid-in-oil nano-suspension which was added to an aqueous solution containing 1% of poly(vinyl alcohol) and maintained at room temperature until total evaporation of the solvent, resulting Ca-P/PHBV nanocomposite microspheres. Tetragonal scaffolds with porosity around 60% were obtained from these nanocomposite microspheres using selective laser sintering. These 3D scaffolds show many advantages related to (i) the nanodimension of the inorganic filler which may provide a better cell response and osteoconductivity, (ii) the nanocomposite microspheres that ensure better dispersion of the nanofiller and (iii) the SLS technique which resulted in a controlled microstructure with totally interconnected pores [56]. Moreover, improved cell proliferation was obtained for Ca-P/PHBV nanocomposite compared to pure PHBV scaffolds.

**Figure 7:**
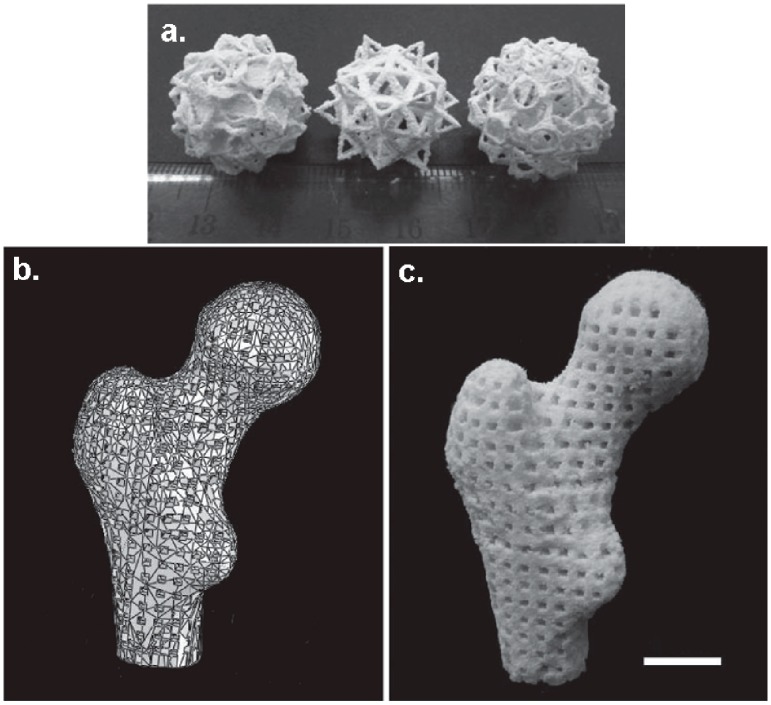
(**a**) Sintered Ca–P/PHBV nanocomposite porous structures based on the following models: salamanders, elevated icosidodecahedron and snarl (from left to right) (**b**) three-dimensional model of a human proximal femoral condyle reconstructed from CT images and then processed into porous scaffold using cubic cells; (**c**) sintered Ca–P/PHBV nanocomposite proximal femoral condyle scaffold. Scale bar, 1 cm. Reproduced with permission from [68].

Porous scaffolds with complex shapes and architecture (Figure 7) were constructed by SLS using Ca–P/PHBV nanocomposite [69]. Moreover, Ca–P/PHBV scaffold representing a human proximal femoral condyle (40% scale-down) was produced by SLS technique. The surface modification of Ca–P/PHBV nanocomposite scaffolds by physically entrapping gelatin and subsequent immobilization of heparin improved the wettability and provided affinity to the growth factor recombinant human bone morphogenetic protein-2 [68]. This osteoconductive nanocomposite with controlled architecture also showed sustained release behavior of osteogenic growth factor and had a great potential for bone tissue engineering [68].

The technique of preparation of Ca-P/PHBV nanocomposite coupled with SLS also offers the possibility of incorporating biomolecules in the nanocomposite microspheres [69]. The advantage of incorporating biomolecules in nanocomposite microspheres is related to the preservation of their biological activity and controlled release. For this purpose, a model protein, bovine serum albumin (BSA), was encapsulated into Ca-P/PHBV nanocomposite microspheres and Ca-P/PHBV/BSA 3D scaffolds with good dimensional accuracy were produced by SLS [69]. It is worth mentioning that the bioactivity of BSA was maintained during SLS processing. In vitro BSA release test showed an initial high activity followed by a slow release of BSA and a slight degradation of the PHBV matrix after 28 days in vitro test [69].

The influence of the SLS process parameters (laser power, scan spacing, layer thickness) on the quality of Ca–P/PHBV nanocomposite scaffolds was also studied [70]. The quality of the scaffolds was quantified by their structure and handling stability, their dimensional accuracy and their compressive properties and the optimized SLS parameters were determined [70].

The most important results regarding the application of 3D-printing techniques to PLA and PHA-based materials are summarized in Table 3.

## 4. Future Perspectives

The use of additive manufacturing methods for the production of artificial organs, tissues or bone implants is an effervescent research area with a promising future. The new era of artificial tissues and organs started twenty years ago with the production of the first 3D FDM printer and since then significant advancements have been made. However, only a few materials have been transferred to mass production and explored with 3D printing and even less of them were found suitable for medical applications. Aliphatic polyesters and, especially, PLA and PHA are suitable materials for in vivo applications due to their biocompatibility, biodegradability, good mechanical strength and processability. The continuous development of new or more specialized biomaterials is often correlated with the progress in the 3D-printing technology enhancing its potential and forcing its rapid development. There are still some challenges in the introduction of 3D-printing technologies as industrial manufacturing tools competing with injection molding and other well-established techniques. They are related to both material and equipment limits, such as reaching high accuracy of the porosity and morphology of the 3D-printed structure according to design specifications, improving the adhesion between layers, fitting the properties and their spatial distribution are some of these challenges. However, the implementation of 3D printing in biomedicine for building prosthetics, tissue grafts and other surgical implants is much more rapid than in other fields. The actual bioprinting technology is suited for the production of artificial organs or implants containing living cells, which requires a sterile environment, but avoiding contamination while handling and keeping the cells alive until they are placed into the patient are still challenges. Likewise, tuning the mechanical and biological properties of artificial tissues and organs is still a challenge and new biocompatible materials are needed to replicate parts of the human body. In addition, it is important for these future materials to be easily combined and manufactured in order to obtain adjustable properties (strength, elasticity, color) for each individual, in respect to its age, gender or race. Besides the aliphatic polyesters presented in this mini-review, some elastomers such as TPU or silicones, which can be processed through different 3D-printing technologies, deserve more attention.

## Figures and Tables

**Table 1:** Mechanical and thermal properties of PLA.

Properties	T_g_, °C	T_m_, °C	Tensile Strength, MPa	Young’s Modulus, GPa	References
PLA (Bio-flex^®^F 6510) solution casting from chloroform	57.5	156.3	15.2	1.17	[28]
PLA (Nature Works^™^ 4032D) solution casting from DMF	-	-	32.8	2.5	[29]
PLA (Nature Works^™^ 4031D) extrusion	-	-	40.9	2.9	[30]
PLA film extrusion grade (Nature Works^™^)	55.3	151.3	40.0	1.4	[31]
PLA (Nature Works^™^ 4032D) Melt compounding	60.0	167.0	40.0	2.7	[32]

DMF—dimethylformamide

**Table 2:** Mechanical and thermal properties of some PHA.

Properties	T_g_, °C	T_m_, °C	Tensile Strength, MPa	Young’s Modulus, GPa	Reference
PHB (Biocycle)—Ccompression molding		164/174	43	3.5	[60]
PHB—solution casting from chloroform			28	2.1	[61]
PHBV 12 mol% HV (Metabolix Inc)—solvent casting from DMF		140	17		[59]
PHBV 12 mol% HV (Metabolix Inc)—solvent casting from DMF	~0	140/154	14	0.8	[62]

**Table 3:** Summary of 3D-printed PLA-based materials.

Technique	Material	Results	Application	Reference
FDM	PLA	Controllable porosity and pore size by controlling the extrusion and 3D-printing parameters	quantifying anisotropic responses of PLA parts	[33]
FDM	PLA	The 3D-printed samples supports the growth of human fetal osteoblast	Bone reconstruction	[39]
FDM	PLA	The 3D-printed model with optimized design displayed a reduction with 62% of the weight as compared to the initial model	Prosthetic foot	[42]
FDM	PLA	Accurate anatomic aspect, reduced amount of raw material, inexpensive final product	Artificial ear	[43]
FDM	PLA, PLA/ionic liquid (IL)	The addition of IL led to enhanced conductivity	Electronic devices	[45]
FDM	PLA/HA	Good dispersion of the HA in the PLA matrix; increased viscosity and compressive modulus for the composites with 15 wt.% HA	Molar tooth	[48]
FDM	PLA, PLA/graphene	Enhanced electrical resistivity and mechanical strength	Electronics	[46]
FDM	PLA	The increased surface roughness and hydrophilicity conducted to cells attachment and proliferation	Bone regeneration	[12]
FDM	TPU/PLA/GO	0.5 wt.% GO led to the highest tensile modulus and cell proliferation	Tissue engineering scaffolds	[51]
FDM	PHA, PHA-g-MA, PHA/palm fibers, PHA-g-MA/ wood flower	Silane treatment of the palm fibers enhanced the adhesion with the polymer matrix; increased mechanical properties and higher degradation rate of the treated composites as compared to pure PHA and untreated composites; Increased tensile strength and antibacterial activity for PHA-g-MA/ wood flower		[63,64]
SLS	PHB	Fidel replication of the 3D-printed structure with the design model; no thermal degradation of the PHB observed after 3D printing	Tissue engineering	[66,67]
SLS	PHBV/Ca-P	The addition of the inorganic filler led to improved cell proliferation; the SLS process didn’t influenced the bioactivity of the incorporated model protein	Bone tissue	[56,68,69]
